# Oxytocin dysfunction in severe mental illnesses following adverse childhood experiences: a systematic review

**DOI:** 10.1186/s12888-026-08279-4

**Published:** 2026-06-22

**Authors:** Guo Guo, Syeda Zoha Fatima Naqvi, Naresh K. Hanchate

**Affiliations:** Genetics and Genomic Medicine Research and Teaching Department, UCL Great Ormond Street Institute of Child Health, University College London, 30 Guilford Street, London, WC1N 1EH UK

**Keywords:** Severe mental illness, Adverse childhood experiences, Oxytocin, OXTR, Single nucleotide polymorphisms

## Abstract

**Background:**

Adverse childhood experiences (ACEs) are recognized as a transdiagnostic risk factor for developing severe mental illnesses (SMIs), including schizophrenia (SCZ), major depressive disorder (MDD), or bipolar disorder (BD). However, the specific associations and underlying mechanisms linking ACEs and SMIs remain unclear. In recent years, oxytocin (OXT), a neuropeptide known for its role in social bonding and stress regulation, has emerged as a crucial pathway linking SMIs and ACEs. This study aimed to investigate the relationship between SMIs among individuals with a history of childhood adversity and oxytocin dysregulation both at biochemical and genetic levels.

**Method:**

We conducted a systematic review assessing OXT measurements or the role of OXT receptor gene (*OXTR*) polymorphisms in individuals with or without SMIs and a history of ACEs. A comprehensive search of four databases (PsycINFO, MEDLINE, Web of Science, and PubMed) was undertaken, adhering to PRISMA (Preferred Reporting Items for Systematic Reviews and Meta-Analyses) guidelines and the review protocol was registered in PROSPERO: CRD42024555819. Comparisons were made between SMI groups and healthy controls (HCs). Data were extracted and appraised independently by two reviewers using the Mixed Methods Appraisal Tool (MMAT). Primary outcomes included group differences in ACE scores, a measure that reflects the type, quantity, and severity of childhood trauma. We also compared group differences in OXT levels, genotype frequencies of *OXTR* single-nucleotide polymorphisms (SNPs), thereby, exploring links between ACEs, OXT-OXTR signalling, and SMIs.

**Results:**

Of 545 reports identified by the search, 14 studies with 5,624 participants met the inclusion criteria. Most studies (*n* = 13; 92.86%) reported that individuals with SMIs exhibited significantly higher ACE scores than HCs. Among the nine of fourteen studies that measured OXT levels, 66% (n = six of nine) reported lower OXT levels in SMIs irrespective of the subgroups, including in SCZ and borderline personality disorder (BPD). Several polymorphisms in *OXTR* were found to have a modulatory effect on SMI outcomes. A subset of SNPs conferred susceptibility, whereas others served as protective factors.

**Conclusion:**

This review highlights associations between OXT system and SMIs with a history of ACE.

**Supplementary Information:**

The online version contains supplementary material available at https://doi.org/10.1186/s12888-026-08279-4.

## Introduction

Severe mental illnesses (SMIs) are psychiatric conditions that significantly affect an individual’s mental, behavioural, physical, and emotional well-being, leading to substantial impairments in daily functioning [1, 2]. These include bipolar disorder (BD), borderline personality disorder (BPD), posttraumatic stress disorder (PTSD), major depressive disorders (MDD), and schizophrenia (SCZ). Individuals with SMIs are at greater risk of poor physical health and experience premature mortality at a significantly higher rate, often 10–20 years earlier than the general population [3]. These psychiatric conditions are unified by shared vulnerability to stress, trauma-related neurobiological changes, emotional and cognitive dysregulation, and overlapping genetic and developmental risk—manifesting along a psychopathology continuum rather than as discrete diseases [4].

Over the past three decades, studies have established adverse childhood experiences (ACEs) as a key environmental risk factor for SMIs [5]. ACEs refer to traumatic events occurring before the age of 18, which include abuse (physical, emotional, and sexual), neglect (physical and emotional), and various forms of household dysfunction [6]. Studies have consistently demonstrated strong associations and a dose-response relationship between ACEs and a wide range of mental health disorders [7–9], increased risk of suicidal behaviour [10, 11], substance abuse [12], and a range of chronic physical health conditions, including cardiometabolic disease [13], metabolic syndrome [14], and myocardial infarction [15]. Emerging evidence also support association between ACEs and adult multimorbidity [16] (two or more chronic conditions). Studies suggest that ACEs can disrupt neurobiological and psychological development, increasing vulnerability to SMIs later in life [7]. However, identifying the underlying causal mechanisms that mediate ACE-linked development of SMIs later in adulthood has been challenging.

Emerging evidence suggests that disruptions in the neurobiological pathways regulating the oxytocin (OXT) system may serve as a critical mechanism linking ACEs to the onset of SMIs [17]. OXT, often referred to as the “love hormone” or “bonding hormone”, is a neuropeptide hormone produced mainly in the hypothalamus in the brain. It acts via the oxytocin receptor (encoded by *OXTR*) and plays a crucial role in a range of social, behavioural, and physiological functions [18]. OXT is released in the brain to regulate neurobiological pathways associated with social bonding (mother-infant, maternal behaviour, and pair bonding) [19], social recognition and affiliation, social behaviours and modulates fear and stress responses. It is also released into the bloodstream at the posterior pituitary to regulate neuroendocrine functions, including reproduction and childbirth [20], milk production [21], cardiovascular [22] and metabolic effects [23]. Given its central role in governing social well-being, dysregulation of the OXT has been associated with psychiatric disorders [24]. Exposure to childhood adversity is inversely correlated with OXT levels measured in plasma [25] and cerebrospinal fluid [26]. These studies suggest that the OXT pathway could be a potential link between ACEs and SMIs [27].

In addition to OXT dysregulation, changes in OXTR can also modulate OXT functioning. These can include OXTR gene variants that can influence changes in gene expression levels or changes in the receptor conformation that can alter binding affinity to its ligand or intracellular signalling mechanisms downstream of OXTR. Indeed, a growing body of evidence suggests that OXTR plays a role in regulating social behaviour [24]. Furthermore, variations in the *OXTR* have been associated with mental health disorders and their implications in social behaviours and emotional regulation [28].

The hypothalamic–pituitary–adrenal (HPA) axis governs physiological responses to stressors [29, 30]. Stressor-induced activation of HPA axis results in increases in circulating levels of glucocorticoids. Prolonged activation of the HPA axis during sensitive periods of early development (i.e., ACEs) can result in higher levels of glucocorticoids for extended durations that can disrupt brain maturation and in turn result in dysregulation of HPA‑axis [31]. Multiple systematic reviews have demonstrated a robust association between childhood maltreatment and alterations in HPA‑axis activity, most notably blunted stress reactivity [32, 33]. Such HPA‑axis dysregulation is a characteristic feature of SMI [34]. HPA axis and oxytocin systems are tightly coupled stress-regulatory systems [35]. OXT dampens the HPA axis potentially acting as an anxiolytic and anti-stress factor, whereas acute activation of HPA axis stimulates OXT release but chronic activation of HPA axis suppresses OXT levels and can downregulate OXTR expression levels in the brain [35].

We reasoned that a dysregulated HPA axis combined with psychological manifestations of ACEs can have severe impacts on OXT system possibly via downregulation of OXT synthesis or reduction in OXTR expression in the brain. Despite growing interest in the role of OXT system in mental health, to our knowledge, no systematic review has comprehensively synthesized evidence on the interplay between ACEs, the OXT system and SMIs. This study aims to address this gap by investigating whether (1) ACE-linked SMIs exhibit dysregulated OXT levels, and (2) *OXTR* variations exacerbate or protect ACE-linked development of specific SMIs compared to healthy controls. Thereby, it seeks to identify the potential associations between OXT system and ACE-induced SMIs.

## Methods

### Overview

The study aimed to systematically review the literature to identify reports investigating circulating levels of OXT or *OXTR* polymorphisms in individuals exposed to adverse childhood experiences (ACEs) with or without a diagnosis of severe mental illnesses (SMIs). This systematic review followed the Preferred Reporting Items for Systematic Reviews and Meta-Analyses (PRISMA) guidelines and is registered with PROSPERO (CRD42024555819).

### Search strategy and selection criteria

A comprehensive search strategy was developed to identify relevant studies published in peer-reviewed journals between 1995 and February 2026. Four electronic databases—PsycINFO, MEDLINE, Web of Science, and PubMed—were searched between February 2024 and February 2026 using the following search terms that included phrases and Boolean operators (“OR” and “AND”). Search terms for individual concept were built using synonyms (for example, for OXT-OXTR, we used “oxytocin” OR “OT” OR “OXT” OR “endogenous oxytocin” OR “oxytocin levels” OR “oxytocin receptor” OR “OXTR” OR “OXTR” OR “OXTR variations” OR “OXTR polymorphisms”) and combined with other concept with “AND” (for example, “ACE” AND “OXT” AND “schizophrenia”). Complete list of search terms are listed in Supplementary Table 1. To ensure the inclusion of all relevant studies, reference lists of included articles were manually screened, and the searches were re-run in February 2026 before the final analysis.

### Inclusion and exclusion criteria

Studies were eligible for inclusion if they met the following specific criteria. First, they needed to include individuals with a history of ACEs, irrespective of a diagnosis of SMI. Studies with a diagnosis of major psychiatric conditions, including SCZ, BPD, BD, PTSD, MDD, generalized anxiety disorder (GAD), and early psychosis, which have a shared vulnerability to stress and trauma, cognitive and emotional impairments, and overlapping genetic and developmental risks. Eligible studies must have also measured OXT levels or analyzed *OXTR* polymorphisms, with comparisons made between individuals exposed to ACEs and diagnosed with SMI and healthy controls (HCs) with a history of ACEs, but no diagnosis of SMI. The primary outcomes of interest included differences in OXT levels, *OXTR* variations, and potential pathways linking OXT system dysregulation with ACEs and SMIs. There were no restrictions on study design due to the absence of randomized controlled trials addressing these variables. Therefore, cross-sectional and cohort studies were included.

Studies were excluded if they did not include individuals exposed to ACEs or if a suitable control group without SMI was absent. Studies that failed to measure OXT levels, analyze *OXTR* polymorphisms, or perform group comparisons based on ACE exposure were also excluded. Research with outcomes unrelated to ACEs, OXT levels, or *OXTR* variations was deemed ineligible. Additionally, reviews, dissertations, grey literature, conference abstracts, preprints, commentaries, non-academic papers, and books or book chapters were excluded. Non-English studies, animal studies, and research focusing on extreme childhood trauma, such as wartime experiences, were also excluded.

After removing duplicates, two independent reviewers (G.G. and S.N.) screened titles and abstracts for eligibility. Articles that passed this initial screening were then assessed (by G.G. and S.N.) for full-text eligibility. Any disagreements at either stage was resolved through discussion, or an additional reviewing author (N.K.H.) was consulted if needed.

### Outcomes

The primary outcome of this review was to examine the associations between ACEs, OXT system dysregulation (including OXT levels and *OXTR* polymorphisms), and susceptibility to SMIs. Specifically, the review investigated differences in OXT levels and *OXTR* variations between individuals with SMIs and HCs.

### Data extraction

Data extraction was conducted systematically to ensure consistency and comprehensiveness. Extracted information included study characteristics such as the first author, year of publication, and country of origin. Data on the study population included sample size, mean age with standard deviation (M ± *SD*), and gender ratio (% female). Information regarding the type of SMI diagnosis was also recorded. Exposure details, including the history of ACEs, OXT levels, and *OXTR* polymorphisms, and sample types used for OXT measurements (blood or serum or others) were extracted. The associations between ACEs, OXT levels, *OXTR* variations, and potential mediating pathways were documented. Data extraction was performed independently by G.G. and reviewed by S.N. to ensure accuracy. Any discrepancies were resolved through discussion and, if necessary, through consultation with an additional reviewing author (N.K.H.).

### Data analysis and quality assessment

The extracted data were synthesized to compare OXT levels, *OXTR* polymorphisms, and ACE exposures between individuals with SMIs and HCs. Categorical data, including genotypic frequencies of specific *OXTR* polymorphisms, were analysed using chi-squared tests. Effect sizes were reported alongside *p*-values to highlight the strength and statistical significance of the observed associations. Mediation analyses were conducted in studies exploring pathways linking ACEs and SMIs to test whether OXT levels acted as mediators. Path coefficients, *b*-values, and confidence intervals were reported to demonstrate the significance and magnitude of these mediation effects. Subgroup analyses were performed based on the type of SMI diagnosis, including SCZ and BPD, to explore variations in OXT levels and *OXTR* polymorphisms. Single-nucleotide polymorphisms (SNPs), such as rs2254298 and rs53576, were examined as potential risk or protective factors.

The methodological quality of the included studies was evaluated using the Mixed Methods Appraisal Tool (MMAT) [36]. The tool assessed five domains related to study design, measurement validity, and confounding variables. Each domain was rated as “Yes,” “No,” or “Can’t tell,” with an overall percentage score calculated for each study. There is no strict cutoff in MMAT for defining poor-quality studies. However, for this review, studies that met only two or fewer criteria were deemed poor and were excluded from the final synthesis. Quality evaluations were performed independently by G.G. and reviewed by S.N. to ensure accuracy. Any discrepancies were resolved through discussion and, if necessary, consultation with the third reviewer (N.K.H.).

## Results

### Literature searches and characteristics of included studies

**Fig. 1:**
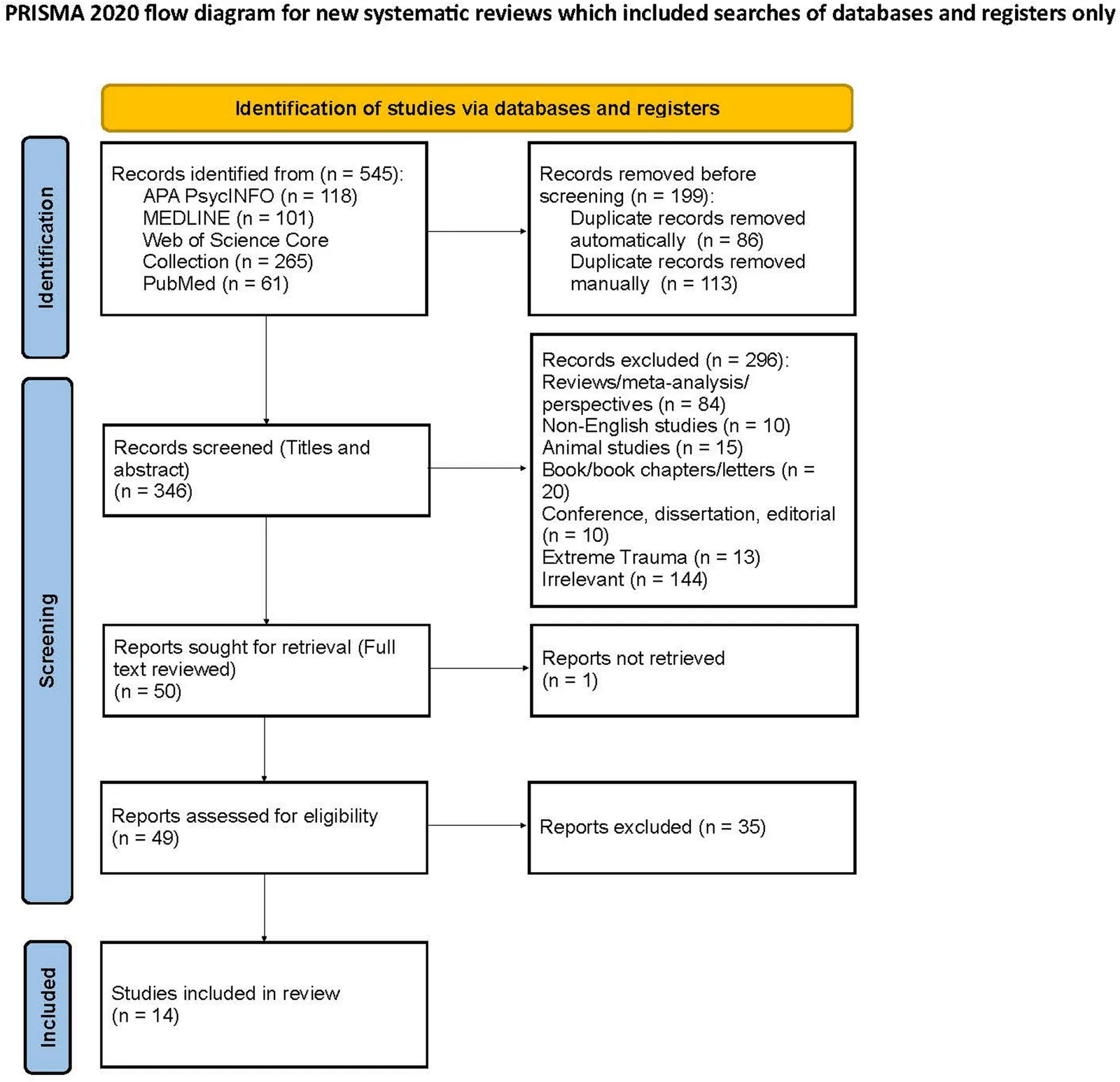
PRISMA 2020 flow diagram for new systematic reviews which included searches of databases, adapted from Page et al., (2021) [37]

Our search identified 545 articles, of which 14 studies met the inclusion criteria and were included in this systematic review (Fig. 1). These studies collectively involved 5,624 participants aged 12 to 54 years and were conducted in seven countries across Asia, Europe, and North America. Most studies employed a cross-sectional design (*n* = 13; 92.86%), while one used a cohort design. Participants varied in gender representation, with five studies including males and females, eight focusing on females only, and one on males only. The SMIs examined comprised SCZ (*n* = 3; 21%), BPD (*n* = 7; 50%), generalized anxiety disorder (GAD) (*n* = 2; 14%), and one each (7%) on major depressive disorders (MDD) and PTSD (Table 1). Although bipolar disorder (BD) is one of the most widely studied SMI, our search strategy did not yield any studies investigating the associations between OXT and ACE-linked BD.

**Table 1:** Summary characteristics of included studies

ID	(Author, Year)	Country	SMI type	Sample size		Age (years) (M ± SD)		Gender ratio (% female)		OXT / OXTR specimen
				SMI	HCs	SMI	HCs	SMI	HCs	
***OXT levels (n = 8)***										
1	(Chen et al., 2024) [38]	China	SCZ	160	80	42.40 ± 9.40	43.45 ± 9.99	40%	42.50%	Plasma
2	(Mielke et al., 2023) [39]	Germany	BPD	131	124	24.21 ± 9.29	14.67 ± 1.53	100%	100%	Plasma
3	(Uzun et al., 2022) [40]	Turkey	GAD	56	28	14.27 ± 1.77	11.60 ± 5.78	57.14%	53.57%	Plasma
4	(Kartal et al., 2022) [41]	Turkey	BPD	31	31	23.06 ± 5.42	25.45 ± 3.74	100%	100%	Blood
5	(Speck et al., 2019) [42]	Germany	SCZ	35	35	40.40 ± 8.80	36.00 ± 10.40	34.29%	34.29%	Plasma
6	(Bomann et al., 2017) [43]	Denmark	BPD	18	20	29.70 ± 9.50	29.50 ± 9.20	100%	100%	Serum
7	(Jobst et al., 2016) [44]	Germany	BPD	21	20	29.85 ± 7.46	30.42 ± 10.55	100%	100%	Plasma
8	(Bertsch et al., 2013) [45]	Germany	BPD	34	40	24.30 ± 5.60	24.60 ± 4.10	100%	100%	Plasma
***OXTR variations (n = 5)***										
9	(Lee et al., 2022) [46]	United States	PTSD	135	93	30.80 ± 6.50	31.0 ± 5.60	100%	100%	Saliva
10	(Zhang et al., 2020) [47]	China	BPD	342	423	33.86 ± 9.03	33.22 ± 8.81	0%	0%	Blood
11	(Flasbeck et al., 2018) [48]	Germany	BPD	148	154	27.70 ± 7.90	24.6 ± 5.40	100%	100%	Saliva
12	(Tollenaar et al., 2017) [49]	Netherlands	MDD, GAD	2180	387	23.06 ± 5.42	25.45 ± 3.74	100%	100%	Blood and saliva
13	(Cristobal-Narvaez et al., 2017) [50]	Spain	Early-psychosis	96	242	40.40 ± 8.80	36.00 ± 10.40	34.29%	34.29%	Buccal mucosa and blood
***OXT levels + OXTR variations (n = 1)***										
14	(Goh et al., 2024) [51]	China	SCZ	382	178	43.87 ± 9.51	42.84 ± 10.07	42.41%	48.31%	Plasma

SCZ = Schizophrenia, BPD = Borderline personality disorder, GAD = Generalized anxiety disorders, PTSD = Posttraumatic stress disorder, MDD = Major Depressive Disorder/ Depression

The quality of fourteen studies included in this review were appraised using the MMAT [36]. Studies were rated from 1 to 5 points; 4 studies scored 5, 7 scored 4, and 3 studies scored 3, demonstrating fair-to-good quality (Table 2). Across the 14 studies, ACEs were predominantly measured using the Childhood Trauma Questionnaire (CTQ; five of fourteen) and its short form (CTQ-SF; seven of fourteen). Two studies each have used the Childhood Experiences of Care and Abuse Questionnaire (CECA-Q) and the Life Stressor Checklist (LSC). These instruments effectively captured aggregate ACE scores. However, most studies did not explore the impact of specific ACE types, such as emotional or physical abuse, on SMI risk. This lack of granularity limits deeper insights into the nuanced relationships between individual ACEs and different SMIs.

**Table 2:** Quality assessment of the included studies using the MMAT checklist

ID	Study designs	(Author, year)	S1.	S2.	1.	2.	3.	4.	5.	Level of evidence
***OXT levels (n = 8)***										
1	Cross-sectional	(Chen et al., 2024)	Yes	Yes	Yes	Yes	Can’t tell	Yes	Yes	4 (80%)
2	Cross-sectional	(Mielke et al., 2023)	Yes	Yes	Yes	Yes	Yes	No	Yes	4 (80%)
3	Cross-sectional	(Uzun et al., 2022)	Yes	Yes	Yes	Yes	No	No	Yes	3 (60%)
4	Cross-sectional	(Kartal et al., 2022)	Yes	Yes	Yes	Yes	No	No	Yes	3 (60%)
5	Cross-sectional	(Speck et al., 2019)	Yes	Yes	Yes	Yes	No	Yes	Yes	4 (80%)
6	Cross-sectional	(Bomann et al., 2017)	Yes	Yes	Yes	Yes	No	Yes	Yes	4 (80%)
7	Cross-sectional	(Jobst et al., 2016)	Yes	Yes	Yes	Yes	Yes	Yes	Yes	5 (100%)
8	Cross-sectional	(Bertsch et al., 2013)	Yes	Yes	Yes	Yes	No	Yes	Yes	4 (80%)
***OXTR variations (n = 5)***										
9	Cross-sectional	(Lee et al., 2022)	Yes	Yes	Yes	Yes	No	Yes	Yes	4 (80%)
10	Cross-sectional	(Zhang et al., 2020)	Yes	Yes	Yes	Yes	Yes	Yes	Yes	5 (100%)
11	Cross-sectional	(Flasbeck et al., 2018)	Yes	Yes	Yes	Yes	No	No	Yes	3 (60%)
12	Cohort	(Tollenaar et al., 2017)	Yes	Yes	Yes	Yes	Yes	Yes	Yes	5 (100%)
13	Cross-sectional	(Cristobal-Narvaez et al., 2017)	Yes	Yes	Yes	Yes	Yes	No	Yes	4 (80%)
***OXT levels + OXTR variations (n = 1)***										
14	Cross-sectional	(Goh et al., 2024)	Yes	Yes	Yes	Yes	Yes	Yes	Yes	5 (100%)

S1. Are there clear research questions? S2. Do the collected data allow to address the research questions? (1) Are the participants representative of the target population? (2) Are measurements appropriate regarding both the outcome and intervention (or exposure)? (3) Are there complete outcome data? (4) Are the confounders accounted for in the design and analysis? (5) During the study period, is the intervention administered (or exposure occurred) as intended?

### Association between ACEs and SMIs

It is well established that there is a strong relationship between ACEs and SMI. Consistent with previous literature, most studies (*n* = 13; 92.86%) reported that individuals with SMIs had significantly higher ACE scores than HCs (Table 3). Only one study, conducted by Uzun et al. (2022) [40], found no significant difference between the SMI group and HCs (*t*= 1.32, *p* = 0.19). This may be attributed to the younger participants in this study (an average of 14.27 years compared to an average of 31.49 years in the other 13 studies). It is possible that adolescence represents a critical period for OXT system maturation, and the immediate effects of ACEs may differ compared to adult populations. ACE-linked SMIs predominantly include female participants, with an average of 72.01% females and 27.99% males. The gender imbalance observed in ACE-linked SMI research may stem from higher exposure of females to multiple ACEs, more complex patterns of ACEs [52], and increased susceptibility to psychiatric symptoms following ACEs [53]. These factors likely contribute to the predominance of female participants in such studies.

**Table 3:** Associations between ACE and SMIs

ID	(Author, Year)	ACE measurement	ACE total scores (M ± SD)				*P value*
			SMI		HCs		
			M	*SD*	M	*SD*	
1	(Chen et al., 2024)	CTQ-SF, structured interviews	60.59	14.18	38.00	4.32	< 0.001
2	(Mielke et al., 2023)	CECA. Q	0.65	0.97	− 0.68	0.58	< 0.01
3	(Uzun et al., 2022)	CTQ	61.62	17.02	56.85	14.69	0.19
4	(Kartal et al., 2022)	CTQ-SF	55.29	17.07	34.06	6.26	< 0.001
5	(Speck et al., 2019)	CTQ-SF	46.00	20.4	35.7	14.3	< 0.05
6	(Bomann et al., 2017)	CTQ	12.60	2.70	6.00	1.30	< 0.05
7	(Jobst et al., 2016)	CTQ	15.00	4.86	6.76	2.35	< 0.001
8	(Bertsch et al., 2013)	CTQ	57.30	17.30	32.20	12.70	< 0.001
9	(Lee et al., 2022)	LSC	50.00	53.80	28.00	20.70	< 0.001
10	(Zhang et al., 2020)	CTQ-SF	51.79	17.53	42.92	15.55	< 0.001
11	(Flasbeck et al., 2018)	CTQ-SF	63.70	19.50	33.90	10.80	< 0.001
12	(Tollenaar et al., 2017)	CTQ-SF, semi-structured CT interview	43.9	14.9	33.40	9.70	< 0.001
13	(Cristobal-Narvaez et al., 2017)	CTQ	41.38	12.91	33.46	8.24	< 0.001
14	(Goh et al., 2024)	CTQ-SF	64.75	24.40	44.63	13.42	< 0.001

CTQ - Childhood Trauma Questionnaire; CTQ-SF - Childhood Trauma Questionnaire short form; CECA.Q - Childhood Experiences of Care and Abuse Questionnaire; LSC - Life Stressor Checklist

### Oxytocin dysfunction in individuals with SMIs

Given that the studies included in this review demonstrated a strong association between ACEs and SMI, therefore, it enabled us to assess whether there are any differences in OXT levels and OXTR polymorphisms between the two populations. Of the 14 studies included, nine (64%) studies measured OXT levels in individuals exposed to ACE with an SMI diagnoses and without an SMI (HCs). The methodology of OXT measurements and the biological specimen type varied between the nine studies. Seven studies measured OXT in plasma and one each in blood and serum. Seven studies used ELISA (enzyme-linked immunosorbent assay) and two used RIA (radioimmunoassay) to measure OXT.

Among these, 77% (seven of nine studies) reported significantly dysregulated OXT in SMI patients compared to HCs. Among these seven studies that reported significant differences in OXT levels between the two groups, 66.6% (n = six of nine studies) reported significantly lower OXT levels in individuals with SMIs compared to HCs and one study reported higher OXT levels (Table 4). The singular study that elevated OXT levels was in younger patients with GAD (average age in this study was 14.27 compared to an average of 31.9 years in the other 13 studies). Importantly, lower OXT levels were found in patients with different SMIs. For instance, out of the six studies that showed lower OXT levels, three each were reported in individuals diagnosed with SCZ and BPD (Table 4). Overall, these studies suggest OXT dysregulation can be common across SMIs. Several studies also investigated the relationship between the impact of ACEs on OXT levels in male and female SMI patients. Of the six studies that reported lower OXT levels in SMI patients, three focused on female patients with BPD, and the three others had a mix of both male and female patients, suggesting ACEs can impact both boys and girls, increasing their susceptibility to SMIs.

**Table 4:** Comparison of OXT levels between SMIs and HCs

ID	(Author, Year)	OXT levels (pg/mL) (M ± SD)				*p value*	Assay
		SMI		HCs			
		M	*SD*	M	*SD*		
1	(Goh et al., 2024)	1160	358	1348	390	< 0.001	ELISA
2	(Chen et al., 2024)	1434	307	1726	449	< 0.001	ELISA
3	(Mielke et al., 2023)	201.94	148.05	255.26	143.12	< 0.01	ELISA
4	(Uzun et al., 2022)	213.71	72.45	166.2	52.70	< 0.001	ELISA
5	(Kartal et al., 2022)	797.38	22.72	835.00	21.98	< 0.001	ELISA
6	(Speck et al., 2019)	4.59	3.35	5.48	4.50	< 0.001	RIA
7	(Bomann et al., 2017)	499.00	224.00	411.00	187.00	0.19	ELISA
8	(Jobst et al., 2016)	421.67 390.97 399.19 403.58	127.70 130.70 142.60 128.54	450.26 471.46 468.19 454.22	204.42 240.63 252.68 233.85	0.59 0.29 0.39	ELISA
9	(Bertsch et al., 2013)	13.90	15.10	30.70	19.80	< 0.001	RIA

ELISA - Enzyme-Linked Immunosorbent Assay; RIA – radioimmunoassay

### Association of *OXTR* polymorphisms with SMIs

OXT exerts its effects by binding to its receptors, OXTR, expressed on cell surface of target cells in brain regions associated with social behaviours, bonding and attachment, and stress-related behaviours [54, 55]. OXTR is also expressed in peripheral metabolic organs, such as the pancreas [56]. Therefore, we next explored the role of *OXTR* single-nucleotide polymorphisms (SNPs) and their association with SMI pathologies. Depending on the location, SNPs can influence OXTR regulation and function in different ways. For instance, SNPs in regulatory elements of *OXTR* can influence its gene expression levels whereas SNPs in the coding region can influence stability of mRNA, protein levels, or binding affinity to its ligand OXT or alter downstream signalling pathways. Notably, altered OXT-OXTR signalling has also been associated with neurodevelopmental [57] and mental health disorders [58].

*OXTR* is located on chromosome 3 in humans at the cytogenetic band 3p25.3 [28]. It belongs to the G-protein-coupled receptor family and spans about 25 kilobases with four exons and three introns, In this review, six of fourteen studies (42%) reported eight single-nucleotide polymorphisms (SNPs) in *OXTR* and their associations with ACE-linked SMIs (Table 5). These studies report that specific genotypes at these eight loci conferred either risk or protection against SMIs (shown in red or green, respectively; Table 5). For instance, the GG genotype of two *OXTR* SNPs (rs237885 and rs53576) appears to increase susceptibility to SMIs, whereas the TT or AA genotype of these variants served as a protective factor, respectively. Similarly, different genotypes at other loci served as either risk or protective factors, suggesting these variants could play a crucial role and impact the expression levels of OXTR in specific brain areas, or binding affinity of OXTR to its ligand OXT or activation dynamics of OXTR receptor in converting extracellular into intracellular signal via downstream signalling pathways.

**Table 5:** Comparison of OXTR variations between SMI group and HCs. Table contains only studies with significant group differences in genotypic frequencies

ID	(Author, year)	SMI	SNP	Risk alleles	Genotype	Genotypic frequencies (*n* (%))		Group differences in genotype frequencies	
						SMI	HCs	*c* ^*2*^	*p*
1	(Goh et al., 2024)	SCZ	rs1042778	G/T	**GG**	220 (57.6)	123 (69.1)	10.217^**^	0.006
					*GT*	128 (33.5)	50 (28.1)		
					*TT*	34 (8.9)	5 (2.8)		
			rs2254298	G/A	**GG**	130 (34.0)	76 (42.7)	12.161^**^	0.002
					**GA**	168 (44.0)	84 (47.2)		
					*AA*	84 (22.0)	18 (10.1)		
			rs237885	G/T	*GG*	30 (7.9)	6 (3.4)	10.490^**^	0.005
					*GT*	155 (40.6)	56 (31.5)		
					**TT**	197 (51.6)	116 (65.2)		
			rs237887	G/A	**GG**	127 (33.2)	89 (50.0)	15.827^***^	< 0.001
					*GA*	186 (48.7)	71 (39.9)		
					*AA*	69 (18.1)	18 (10.1)		
			rs237895	C/T	*CC*	33 (8.6)	13 (7.3)	12.459^**^	0.002
					*CT*	160 (41.9)	49 (27.5)		
					**TT**	189 (49.5)	116 (65.2)		
			rs237899	G/A	**GG**	187 (49.0)	111 (62.4)	9.763^**^	0.008
					*GA*	151 (39.5)	56 (31.5)		
					*AA*	44 (11.5)	11 (6.2)		
			rs53576	G/A	*GG*	67 (17.5)	13 (7.3)	11.025^**^	0.004
					**GA**	155 (40.6)	75 (42.1)		
					**AA**	160 (41.9)	90 (50.6)		
2	(Zhang et al., 2020)	BPD	rs53576	G/A	**GG**	125 (38.0)	177 (43.2)	6.054^*^	0.048
					**GA**	20 (6.1)	38 (9.3)		
					*AA*	184 (55.9)	195 (47.6)		
			rs237897	G/A	**GG**	121 (37.6)	165 (40.8)	6.754^*^	0.034
					**GA**	14 (4.3)	34 (8.4)		
					*AA*	187 (58.1)	205 (50.7)		
3	(Tollenaar et al., 2017)	MDD, GAD	rs2254298	G/A	**GG**	1912 (87.7)	352 (91.0)	7.252*	0.027
					*GA*	259 (11.9)	31 (8.0)		
					**AA**	9 (0.4)	4 (1.0)		

Table contains only studies with significant group differences in genotypic frequencies

Note: ^*^p < 0.05, ^**^p< 0.01, ^***^p < 0.001. Genotypes previously reported to serve as a risk or protective factor are highlighted in italics or in bold font, respectively

These analyses suggests that specific SNPs in the *OXTR* are associated with susceptibility to various SMIs. For instance, two studies (Goh et al. (2024) [51] and Tollenaar et al. (2017) [49]) investigated the same SNP, rs2254298. Goh et al. (2024) found that the AA genotype was significantly more prevalent in the SCZ group (22.0%) compared to HCs (10.1%) (*χ²* = 12.161, *p* = 0.002) and Tollenaar et al. (2017) reported that the GA genotype of rs2254298 was more frequent in patients with MDD or GAD (11.9%) compared to HCs (8.0%) (*χ²* = 7.252, *p* = 0.027). Similarly, the *OXTR* SNP rs53576 was also reported in two studies. Goh et al. (2024) found that the GG genotype frequency was higher in the SCZ group (17.5%) compared to HCs (7.3%) (*χ²* = 11.025, *p* = 0.004). Zhang et al. (2020) [47] reported that the AA genotype was significantly more common in the BPD group (55.9%) compared to HCs (47.6%) (*χ²* = 6.054, *p* = 0.048).

We next focused on three studies that examined G×E interactions, demonstrating that *OXTR* variations moderate the relationship between ACEs and SMIs. Of these, one study highlighted that high additive genetic risk scores, calculated across multiple SNPs, amplified the impact of ACEs on schizophrenia symptoms (*β* = 0.226, *SE* = 0.031, 95% CI **=** [0.169, 0.290], *p* < 0.001) Goh et al. (2024). Similarly, other studies identified significant ACE × *OXTR* interactions predicting psychotic symptoms (*p* < 0.001) Cristobal-Narvaez et al. (2017) [50], and individuals carrying the A allele of rs53576 (AA or GA genotypes) demonstrated heightened empathy for psychological pain when exposed to ACEs, whereas GG carriers were less affected (*p* = 0.037) [48].

## Discussion

The present systematic review revealed associations between ACEs, OXT system, and SMIs. ACEs are critical environmental factors predisposing individuals to SMIs. Consistent with this notion, our findings revealed that individuals with SMIs had a significantly higher prevalence of ACEs than healthy controls (HCs). Additionally, most studies included in this review report reduced OXT levels in patients with different SMIs, indicating that dysregulation of the oxytocinergic system may play a role in the pathophysiology of SMIs. Finally, subsets of oxytocin receptor (*OXTR*) polymorphisms conferred resilience or susceptibility to SMIs, suggesting that gene-environment interactions affecting OXT system (both biochemically at OXT levels and downstream *OXTR* level) may play a crucial role in the development of SMIs.

ACEs are well-established leading causes of adult morbidity and mortality [59]. ACEs have been associated with higher prevalence rates of mental illnesses, including depression [60], anxiety [61], and PTSD [62], and physical illnesses, such as higher risk of cardiovascular disorders [60, 63], diabetes, and weakened immune function [64]. Consistent with a large body of evidence, our study identified strong associations between ACE exposure and the prevalence of SMIs. Importantly, these associations were observed across all the SMI types reported in our study, including SCZ, BPD, PTSD, MDD, and early psychosis. This indicates that ACEs can have a profound impact on adult mental health and serve as a common underlying factor that increases susceptibility to various SMIs. However, the specific SMI outcomes vary among individuals depending on the ACE type, duration, severity, genetic predispositions, and environmental influences. One key mechanism underlying these variations could be a prolonged activation of stress response systems following ACE exposure. Chronic stress can lead to heightened cortisol levels, which negatively impact brain development and neurobiological pathways involved in social bonding and social behaviours [65].

In recent decades, the oxytocinergic system has emerged as a crucial neurobiological pathway for its role in social behaviours and social bonding [54]. Several studies have shown that its dysregulation is associated with several mental health conditions (such as depression [66]) and neurodevelopmental disorders, such as autism spectrum disorder [57]. Our systematic review revealed that most studies reported reduced oxytocin levels in SMI patients compared to healthy controls. OXT dysregulation reported in this review are consistent with a recent systematic review reporting strong reductions in OXT levels associated with PTSD and emotional trauma ([67]. While these studies focused on emotional trauma and PTSD in mostly adults, our studies extend these findings beyond PTSD experienced during adulthood to ACE-related psychiatric conditions. In this review, multiple studies reported reduced OXT levels in different SMI types (SCZ and BPD). These findings are consistent with previous research highlighting the role of OXT in emotional regulation, social behaviour, and stress response [55]. For instance, prior studies have demonstrated that lower OXT levels are linked to impaired social cognition in SCZ [17] and increased interpersonal dysfunction in BPD [68]. While ACE is a common underlying factor of SMIs in these studies, the genetic makeup of these patients is unknown. It is also plausible that these patients may harbor mutations in other genes associated with social behaviours, brain maturation, or function.

Here, we focused on data available on the variants associated with the oxytocinergic system. In particular, identifying *OXTR* variations as mediators of susceptibility to SMIs adds to the growing body of evidence on gene-environment interactions. However, our findings also underscore the complexity of these relationships, as the protective effects of certain variants (such as the GG genotype) appear to vary depending on the type of ACE, timing, and chronicity. This aligns well with prior studies that reported increased sensitivity to negative parenting among GG carriers, which further adds to the complexity of the role of *OXTR* polymorphisms [69].

This review has several limitations that should be carefully considered. First, the reliance on retrospective self-report tools, such as the CTQ, introduces the risk of recall bias and underreporting of ACEs. While some studies incorporated structured interviews, the lack of multi-method assessments limits the accuracy and reliability of ACE measurements. Second, heterogeneity in methodologies of OXT measurement and sampling different biological specimens [70]. Most studies measured peripheral OXT levels using plasma, which may not accurately reflect central OXT concentrations due to their unstable nature^38^. Studies also used different methodologies (ELISA and RIA) for OXT measurements which differ in sensitivities and detection ranges, which can make it challenging to make comparisons between studies. However, a recent systematic review of the literature suggests a positive correlation between central and peripheral levels of OXT during stressful conditions or intranasal OXT delivery, but not in baseline conditions [71]. Third, lack of longitudinal OXT measurements (before, during, and after ACEs), makes it challenging to associate if the decrease in OXT measurements is due to ACE or of developmental origins. It is plausible that individuals with SMIs and lower OXT had pre-existing developmental issues that affected hypothalamic structures due to their genetic ‘makeup’ that increased vulnerabilities to ACE-induced HPA axis dysregulation and OXT-OXTR development, function, or its regulation. ACEs could affect the developing brains and further increasing vulnerabilities to SMI psychopathology. Longitudinal studies with multiple time-point measurements are necessary to establish temporal relationships and assess how OXT levels or *OXTR* variations evolve over time in response to ACEs. Finally, candidate-gene based association is debated in psychiatric conditions [4]. It is therefore highly likely that multiple gene loci or variants in genes associated with development and function of OXT-producing neurons or OXT synthesis or OXTR expression could be involved in ACE-associated development of SMIs. 

Our systematic review is composed mostly of cross-sectional design, which contain data obtained at a single time point and thus often hinder causal inferences (cause-and-effect relationships) between ACEs, OXT system, and SMIs. Additionally, studies on BPD included in this review mostly involve female participants, who are often underrepresented in research. This observation highlights that are females are more likely to experience more forms of ACEs and that they are highly susceptible to ACE-associated SMIs. The small sample sizes in most included studies limit statistical power, particularly in analyses of *OXTR* polymorphisms. Publication bias may have influenced the findings, as studies with null results are less likely to be published. Additionally, the relationship between ACEs and OXT dysregulation in other major SMIs, such as bipolar disorder, remains understudied.

From a clinical and policy perspective, this review highlights the importance of early identification and prevention of childhood adversity to reduce the risk of SMIs. Strategies aimed at promoting safe, stable, and nurturing environments for children, as recommended by the Centers for Disease Control and Prevention [72], should be prioritized in public health initiatives. Moreover, OXT-related biomarkers, such as peripheral OXT levels and *OXTR* genotypes, hold promise as tools for identifying individuals at heightened risk of SMIs, potentially informing targeted interventions. While therapies such as intranasal OXT administration have shown promise in reducing stress-related symptoms [17], careful consideration of context-dependent effects and potential risks is essential to ensure safety and efficacy.

In conclusion, this review highlights the interplay between ACEs, OXT system dysregulation, and susceptibility to SMIs, shedding light on the biological mechanisms underlying ACE’s long-term impact on mental health.

## Supplementary Information

Below is the link to the electronic supplementary material.

Supplementary Material 1

## Data Availability

No datasets were generated or analysed during the current study.
