# Supplementary material for: Oxytocin dysfunction in severe mental illnesses following adverse childhood experiences: a systematic review
Source: BMC Psychiatry. 2026 Jun 22;26:753. doi: 10.1186/s12888-026-08279-4 (PMC13628910; doi:10.1186/s12888-026-08279-4)
Supplement: Supplementary file 1 [file 12888_2026_8279_MOESM1_ESM.docx]

Supplementary information

| Supplementary table 1. Search strategy used for mining databases | |
| --- | --- |
| Childhood and adolescence | (“child” OR “childhood” OR “children” OR “preschool” OR “school age” OR “early life” OR “youth” OR “young people” OR “preadolescent” OR “adolescent” OR “adolescence” OR “teen” OR “minor” OR “juvenile”) |
| AND | |
| Adversity | (“adversity” OR “childhood adversity” OR “adverse experience” OR “adverse childhood experiences” OR “ACEs” OR “early life stress” OR “ELS” OR “early life adversity” OR “ELA” OR “trauma” OR “early life maltreatment” OR “ELM” “psychological stress” OR “psychological trauma stress” OR “environment stress” OR “social stress” OR “chronic stress” OR “maltreatment” OR “abuse” OR “physical abuse” OR “sexual abuse” OR “rape” OR “emotional abuse” OR “domestic violence” OR “neglect” OR “physical neglect” OR “emotional neglect” OR “bully” OR “household dysfunction” OR “substance abuse” OR “mental illness” OR “domestic violence” OR “incarceration” OR “parental separation” OR “parental loss” OR “economic hardship”) |
| AND | |
| Oxytocin | (“oxytocin” OR “OT” OR “OXT” OR “endogenous oxytocin” OR “oxytocin levels” OR “oxytocin receptor” OR “*OXTR*” OR “*OXTR* variations” OR “*OXTR* polymorphisms”) |
| AND | |
| Severe mental illness | (“serious mental illness” OR “SMI” OR “severe mental illness” OR “psychotic disorder” OR “psychosis” OR “schizophrenia” OR “delusional disorder” OR “schizoaffective disorder” OR “anxiety disorder” OR “posttraumatic stress disorder” OR “PTSD” OR “major depressive disorder” OR “MDD” OR “major depression” OR “clinical depression” OR “mood disorder” OR “affective disorder” OR “borderline personality disorder” OR “BoPD”) |
